# Accuracy and Reproducibility of Digital Area and Depth Measurements of Surface Wounds: Benchtop and Clinical Validation

**DOI:** 10.3390/diagnostics16071055

**Published:** 2026-04-01

**Authors:** Ron Linden, Perry V. Mayer, Rose Raizman, Hanna Varonina, Laura M. Jones-Donaldson, Danielle Dunham

**Affiliations:** 1 Judy Dan Research and Treatment Centre, Toronto, ON M2R 1N5, Canada; 2 The Mayer Institute, Hamilton, ON L8R 2R3, Canada; drpvmayer@themayerinstitute.ca; 3 Scarborough Health Network, Toronto, ON M1E 4B9, Canada; rraizman@shn.ca; 4 Lawrence S. Bloomberg Facility of Nursing, University of Toronto, Toronto, ON M5T 1P8, Canada; 5 MolecuLight Inc., Toronto, ON M5G 1T6, Canada; hvaronina@moleculight.com (H.V.); ljones@moleculight.com (L.M.J.-D.); ddunham@moleculight.com (D.D.)

**Keywords:** arterial ulcer, AutoDepth, chronic wounds, diabetic foot ulcer, digital planimetry, MolecuLightDX, pressure injury, traumatic wound, venous leg ulcer, wound measurement

## Abstract

**Background/Objectives**: Accurate and reproducible wound measurement is essential for monitoring healing and guiding treatment decisions. Conventional ruler-based techniques are prone to geometric overestimation and operator variability. This study evaluated the accuracy and reproducibility of the MolecuLightDX wound imaging device for measuring wound surface area and depth compared with ruler-based measurements and ground truth digital photography methods. **Methods**: This investigation comprised two companion studies: a prospective, paired, multicenter clinical study comparing MolecuLightDX measurement with the ruler method against an image-based ground truth, and a bench and clinical validation of the AutoDepth feature against a calibrated three-dimensional optical scanner. The area of study included 17 benchtop wound models and enrolled 27 patients (33 wounds; area range: 0.56–23.04 cm^2^) across two wound care centers, and the AutoDepth study included 17 benchtop wound models and 34 clinical wounds (depth range: 0.06–4.13 cm). Accuracy, intra- and inter-user variability, and agreement were assessed using the mean percentage error (MPE), coefficient of variation (CV), intraclass correlation coefficients (ICC), and Bland–Altman analysis. **Results**: The device demonstrated high accuracy and reproducibility for both wound surface area and depth measurements compared with ruler-based and ground truth digital photography methods. The MPE for surface area was <10%, representing a tenfold improvement over ruler estimation (77.9%). For wound area, intra- and inter-user CVs were <10%, and for depth, ICCs were ≈0.99. **Conclusions**: The MolecuLightDX device provides accurate and consistent wound area and depth measurements across diverse wound types, demonstrating superior accuracy and reproducibility compared with conventional ruler-based methods and supporting its integration into wound assessment workflows.

## 1. Introduction

Chronic, non-healing surface wounds affect quality of life (QoL) and limit daily living activities, especially among older adults and people with diabetes or limited mobility [1,2,3]. As prevalence rises with age and comorbidity [2,4,5], consistent and objective wound measurement is important for clinical documentation.

Accurate and consistent wound measurement is essential not only for monitoring wound healing but also for predicting healing outcomes and guiding clinical decisions. Evidence shows that a ≥40% reduction in the wound surface area within 4 weeks is a reliable indicator of eventual complete healing, whereas a slower reduction suggests a stalled or chronic wound [6,7]. Therefore, this relationship highlights that surface area change, rather than simple linear dimensions (i.e., length or width), is a meaningful metric for predicting wound healing progression and treatment efficacy [8]. Traditional standard of care (SOC) wound measurement techniques are variable, unstandardized, and have high error rates, often due to overestimation of the wound surface area [9,10]. The paper-based ruler method is the most commonly used SOC technique [11], which approximates the area by multiplying length and width and requires the clinician to record measurements manually. However, this approach is prone to variability, even when standardized orientation methods like the clock-face convention are used. Because wound shapes are irregular, geometric estimation can introduce measurement variability, potentially masking subtle healing changes and misinforming clinical decisions—particularly when surface area, rather than length or width, is the predictor of wound healing [6,7,8]. The use of digital photography paired with calibration makers and edge tracing software methods are available but are not used in routine practice [12]. Wound depth is an important measure of healing because wounds generally heal from the base upward [13]; thus, accurately assessing the depth provides a meaningful indicator of tissue recovery and progression toward closure [13]. Contact-based wound depth measurements are made using a probe and a ruler [14]; however, there is significant variability and inconsistency related to provider technique [15]. Depth readings depend on probe pressure and angle and require contact with the wound, which can distort the measurement and cause patient discomfort when pressing on sensitive tissues [14,15]. These limitations highlight the need for a standardized, digital wound surface area and depth measurement method that reduces operator dependence and minimizes tissue contact when feasible.

The aim of our research was to determine whether the wound measurement feature of the MolecuLightDX (MolecuLight, Inc., Toronto, ON, Canada) imaging device provides acceptably accurate surface area and depth measurements compared with reference standards.

## 2. Materials and Methods

### 2.1. Study Overview and Design (Companion Investigations)

#### 2.1.1. Study Design

Two companion investigations were conducted to evaluate the measurement accuracy and reproducibility of the MolecuLightDX wound imaging device (MolecuLight Inc., Toronto, ON, Canada). MolecuLightDX is an FDA-cleared handheld Class II medical device that combines fluorescence imaging for detecting elevated bacterial loads with digital wound measurement capabilities. The wound imaging platform also supports thermal imaging and is designed for future integration of additional imaging modalities, enabling comprehensive wound assessment and longitudinal tracking.

**Figure 1:**
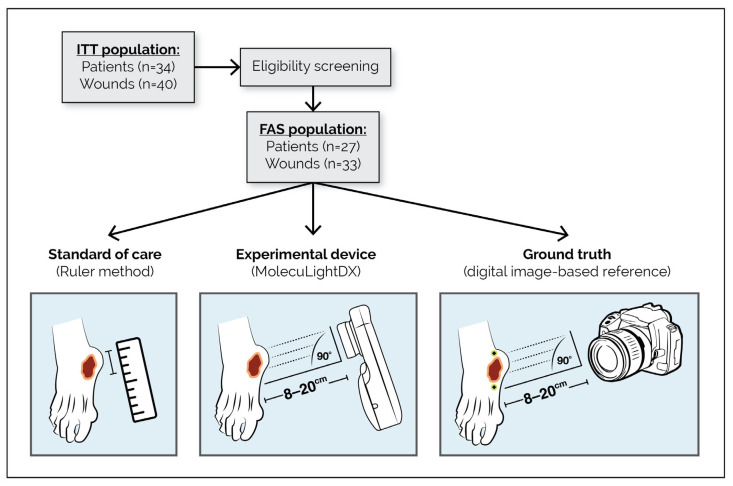
Schematic of wound area measurement study design. Flow diagram outlining patient enrollment and study procedures for evaluating wound surface area measurement accuracy (clinical component). The ITT population included 34 patients (40 wounds); after eligibility screening, 27 patients (33 wounds) formed the FAS. Each wound was assessed using three approaches: the standard of care (ruler method), the experimental device (MolecuLightDX), and a digital image-based ground truth reference. High-resolution reference images were captured with a Canon EOS R10 (Canon Canada, Toronto, ON, Canada) camera positioned 8–20 cm from the wound at a 90° angle, with two 1 cm calibration stickers placed on opposing sides for geometric scaling. Abbreviations: FAS, full analysis set; ITT, intention-to-treat.

The first was a single-arm, prospective, paired, multicenter clinical study comparing wound area measurements obtained using the MolecuLightDX (in both automatic and manual imaging modes) and the conventional ruler method against a digital, image-based ground truth derived from consensus tracing by an expert panel (ClinicalTrials.gov NCT06682923). The objective of this component was to assess the accuracy and precision of wound area measurements captured with the MolecuLightDX compared with the conventional methods. A schematic overview of this area measurement study design is presented in Figure 1.

**Figure 2:**
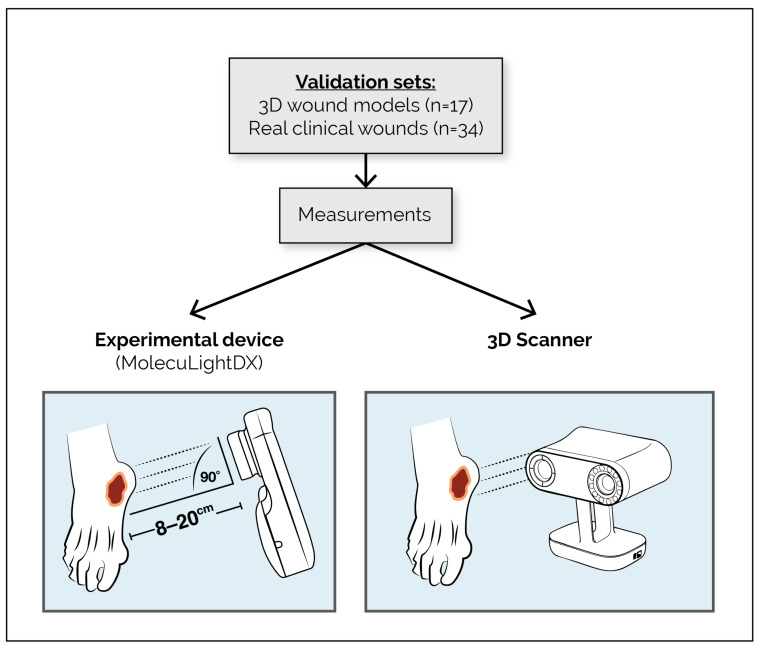
Schematic of AutoDepth (depth measurement) validation study design. Diagram showing validation sets and measurement workflow for evaluating wound depth accuracy. Validation included 17 3D wound models and 34 real clinical wounds. Measurements were obtained using the experimental MolecuLightDX device and an Artec Leo 3D Scanner (Artec 3D, Santa Clara, CA, USA) positioned 8–20 cm from the wound at a 90° angle. Abbreviations: 3D, three-dimensional.

The second investigation was a statistically powered bench and clinical validation study designed to evaluate the accuracy and reproducibility of the MolecuLightDX AutoDepth measurement feature. In this study, depth measurements obtained with the MolecuLightDX were compared with reference values generated using a calibrated three-dimensional (3D) optical scanner, which served as the ground truth standard. A schematic overview of this depth measurement study design is presented in Figure 2.

#### 2.1.2. Eligibility Criterion

For the area measurement study, the inclusion criteria were as follows:

- Patients aged ≥22 years old.
- Patients willing to provide informed consent and comply with all study procedures.
- Wounds included diabetic foot ulcers (DFU), venous leg ulcers (VLU), arterial ulcers (ALU), pressure ulcers (PU), and traumatic wounds.
- Wounds with surface area > 0.5 cm^2^ and well-defined wound borders.

Efforts were made to include variability in patient and wound characteristics, including a range of skin tones (assessed by Fitzpatrick skin type), wound sizes, and wound durations.

The exclusion criteria were as follows:

- Circumferential wounds were excluded because these wounds extend around a limb or body surface and cannot be reliably captured within a single planar image for standardized area measurement.
- Wounds with tunneling or undermining.
- Wounds with dimensions > 18.5 cm in length or >13.5 cm in width, as the device cannot measure wounds larger than these limits.
- Wounds located in difficult-to-measure locations (e.g., curved body surfaces, deep skin folds, the groin, under the breast, or areas with pronounced sacral or buttock curvature).
- Malignant wounds.
- Patients with any contraindication to routine wound assessment (e.g., conditions preventing safe wound exposure or imaging, including malignant wounds, friable wounds, or wounds with active bleeding).

For the AutoDepth measurement evaluation, eligibility criteria were applied at the wound level rather than at the patient level to ensure a representative sample across wound types and depths. Wounds were selected if depth measurement was clinically indicated and suitable for non-contact optical imaging with the MolecuLightDX device. Eligible wounds included DFU, VLU, ALU, and PU, as well as traumatic wounds. Each wound was required to have an open wound bed with visible depth contours (wounds with height were ineligible), clearly defined margins, and no heavy wound exudate or eschar that could interfere with optical depth detection. Wounds with circumferential configuration, excessive tunneling or undermining beyond visible margins, sinus tracts, or those located in areas where maintaining the recommended 8–20 cm imaging distance was not feasible were excluded. Wounds were measured prior to wound care intervention; however, wounds with active bleeding prior to interventions were also excluded. To capture variability in clinical presentation, selection targeted diversity in anatomical location, tissue composition (granulating, sloughy, or mixed), and skin tone diversity documented using Fitzpatrick classification. Each wound was verified by the clinical investigator to ensure that optical imaging could be performed safely and consistently.

#### 2.1.3. Area Measurement Validation Study

To evaluate the accuracy and reproducibility of the MolecuLightDX wound measurement software (Software B.1.2.273) in both manual and automatic imaging modes, 17 benchtop wound models with known dimensions and clearly defined borders were imaged and measured using the MolecuLightDX.

For the benchtop wound models, 17 two-dimensional (2D) benchtop wound models were designed and printed in-house using SolidWorks (Software 2014) computer-aided design (CAD) software to replicate the shapes and sizes of real clinical wounds with known reference values for area, length, and width. These printed 2D models were affixed to one of four types of 3D surfaces: flat surface, cylindrical surface, slanted surface, and cylindrical slanted surface. Five users performed three replicate measurements of each wound model in both manual and automatic imaging modes using the MolecuLightDX device. Measurement images were captured at 8–20 cm from the wound surface, per manufacturer’s guidelines, using a rangefinder on the device. During imaging, the device was positioned approximately parallel to the wound surface, and the integrated illumination system provided standardized lighting conditions to minimize the influence of ambient light. The device also incorporates image-quality checks that detect issues such as glare, excessive imaging angles, or out-of-focus capture to help ensure accurate measurement acquisition. Wound boundaries were identified using the MolecuLightDX device in either automatic or manual mode. In automatic mode, the device’s algorithm automatically detected the wound margins, with optional user adjustment as needed. In manual mode, the device required users to trace the wound edges precisely on the touchscreen interface. Device-calculated measurements were then compared to the true physical dimensions (ground truth) of each model to assess accuracy. Precision was assessed by calculating the intra-user and inter-user repeatability.

##### Clinical Measurement Protocol

Each enrolled wound was measured using three methods: standard of care (ruler method), experimental device method (MolecuLightDX), and the digital image-based reference (ground truth).

Ruler method: Wound length and width were measured with a disposable paper ruler by a wound care clinician. Area was calculated by multiplying the length by the width.

MolecuLightDX: Wounds were imaged using the MolecuLightDX device per manufacturer’s instructions from 8 to 20 cm away. Following image collection, 5 trained clinician users (1 medical doctor [MD], 1 nurse practitioner [NP], 2 registered nurses [RNs], and 1 chiropodist) each performed 3 replicate measurements per wound in both automatic and manual modes, resulting in 6 measurements per clinician per wound. This yielded a total of 15 measurements per wound for each method (30 total), which were analyzed separately and in random order. In manual mode, the same users traced wound borders directly on the device screen. In automatic mode, the users identified the rough location of the wound in the image, and the device’s embedded algorithm automatically generated wound borders, which could be adjusted.

Ground truth: High-resolution images (5328 × 4000 pixels, 24-bit color depth, standard Red–Green–Blue [sRGB] color space, and 72 dots per inch [dpi]) were captured using a Canon EOS R10 (Canon Canada, Toronto, ON, Canada) camera. Two 1 cm calibration stickers were positioned adjacent to and on opposite sides of each wound. Images were downloaded and evaluated offline in random order. Three expert clinicians (1 MD, 1 NP, and 1 chiropodist) independently traced the wound borders using Amadine software (Version 1.8) (BeLight Software Ltd., Odesa, Ukraine). The Simultaneous Truth and Performance Level Estimation (STAPLE) algorithm [16,17] was used to compute a consensus reference area, length, and width for each wound, generating a gold standard that accounted for the varying performance of raters. This represented the ground truth measurement to which the MolecuLightDX and ruler measurements were compared.

#### 2.1.4. AutoDepth Validation Study

To evaluate the software accuracy and reproducibility of the MolecuLightDX AutoDepth measurement capability, a statistically powered validation study using 17 3D wound models (designed in-house and 3D printed or purchased from Vata Inc., Canby, OR, USA) and 34 real clinical wounds was performed. First, the true depths of the wound models and clinical wounds were measured using a calibrated Artec Leo 3D Scanner (Artec 3D, Santa Clara, CA, USA). Measurement images were captured with the MolecuLightDX between 8 and 20 cm from the wound/model surface, per manufacturer’s guidelines, using a rangefinder on the device. To validate the AutoDepth feature of the MolecuLightDX, the wound model depths were measured on the MolecuLightDX in triplicate by three users. The clinical wounds were measured in duplicate by 4 users, also on the MolecuLightDX. Testers were blinded to the true depths.

### 2.2. Study Sites and Population

The clinical component of both the area measurement and AutoDepth investigations was conducted at 2 outpatient wound care centers in Ontario (ON), Canada: the Mayer Institute (Hamilton, ON, Canada) and the Judy Dan Research and Treatment Centre (North York, ON, Canada). The patients attending these wound care centers had a range of wounds of varying etiology, anatomical location, depth, and skin tone.

### 2.3. Outcomes

For area (clinical), the primary endpoints were accuracy of the MolecuLightDX, in both manual and automatic modes, versus the ground truth. The accuracy of ruler measurements versus the ground truth was also assessed. Secondary endpoints included MolecuLightDX versus ruler accuracy, agreement between manual and automatic modes, and inter- and intra-user variability coefficient of variation (CV). For area (bench and clinical validation), pass criteria included mean error for area, length, and width ≤ 10%, and intra-user and inter-user CV < 10%.

For AutoDepth (clinical and bench), endpoints were mean absolute depth error versus the 3D-scanner ground truth and intra- and inter-user intraclass correlation coefficients (ICCs).

### 2.4. Statistical Analysis

The accuracy of wound surface area measurements from the MolecuLightDX device (manual and automatic modes) was evaluated against the ground truth reference using paired t-tests, with comparisons also made to standard ruler-based measurements. Reproducibility was assessed via inter- and intra-user CV, with <10% considered acceptable. Statistical significance was set at α = 0.05 unless otherwise specified. Additional analyses included root mean square error and agreement assessment via Bland–Altman plots using Python (Version 3.13.2). Subgroup and covariate analyses, reported descriptively, explored performance across wound types, sizes, durations, and Fitzpatrick skin types (I–VI) to illustrate applicability across diverse wound and patient characteristics; these were not powered for statistical significance.

Device-calculated depth measurements were compared to the ground truth depth of each model or clinical wound to calculate mean absolute measurement error. ICC for intra- and inter-user variability were calculated using a 2-way random effects ANOVA model. Acceptable AutoDepth accuracy was defined as a mean absolute measurement error of ≤±0.2 cm or ≤10%.

### 2.5. Ethical Approval and Consent

All procedures followed Good Clinical Practice per ISO 14155:2020 [18] and the Tri-Council Policy Statement and were approved by Veritas Independent Review Board Inc. (Montreal, QC, Canada). Patient data and wound assessment results were recorded and de-identified to protect patient confidentiality. Written, informed consent was obtained from all participants, including for the publication of photographs or images.

## 3. Results

### 3.1. Patients

For the wound area measurement investigation, 17 wound models were used for benchtop testing, and a total of 34 patients (40 wounds) were enrolled for the clinical component. The FAS included 27 patients with 33 wounds after the exclusion of seven patients with seven wounds that did not meet the eligibility criteria. The FAS comprised seven females and 20 males, aged between 42 and 90 years. Wound locations included the ankle (*n* = 12), forefoot (*n* = 8), midfoot (*n* = 6), shin or calf (*n* = 4), hip (*n* = 2), and knee (*n* = 1). Wound types were DFU (*n* = 12), VLU (*n* = 9), ALU (*n* = 3), PU (*n* = 4), and traumatic wounds (*n* = 5). Wound areas were <2 cm^2^ (*n* = 13), 2–10 cm^2^ (*n* = 14), and >10 cm^2^ (*n* = 6). Wound duration was <12 months in 20 patients and >12 months in 13 patients. Fitzpatrick skin type categories were represented as follows: type I (*n* = 5), type II (*n* = 5), type III (*n* = 9), type IV (*n* = 4), type V (*n* = 2), and type VI (*n* = 2). Ground truth reference areas for the benchtop wound models ranged from 2.78 cm^2^ to 91.9 cm^2^ and for clinical wounds from 0.56 cm^2^ to 23.04 cm^2^.

The AutoDepth validation study included 17 wound models and 34 images of real wounds, encompassing DFU (*n* = 15), VLU (*n* = 7), PU (*n* = 11), and one surgical site wound (*n* = 1). Ground truth reference depths for the benchtop models ranged from 0.13 cm to 2.78 cm and for clinical wounds from 0.06 cm to 4.13 cm.

### 3.2. Wound Area Measurement

The predefined maximum acceptable error of 10% was achieved for both of the MolecuLightDX imaging modes. When testing the benchtop models, the mean percentage error was 2.57% (standard deviation [SD]: 0.023; 95% confidence interval [CI]: 2.27–2.87) for the automatic mode and 3.34% (SD: 0.024; 95% CI: 3.04–3.64) for manual mode (Table 1). Inter-user median CVs were 0.012 (95% CI: 0.010–0.013) for the automatic mode and 0.022 (95% CI: 0.021–0.024) for the manual mode, while intra-user mean CVs were 0.017 (95% CI: 0.015–0.018) and 0.031 (95% CI: 0.030–0.033), respectively (Table 1). All mean CV values were below the acceptance threshold of 0.10.

**Figure 3:**
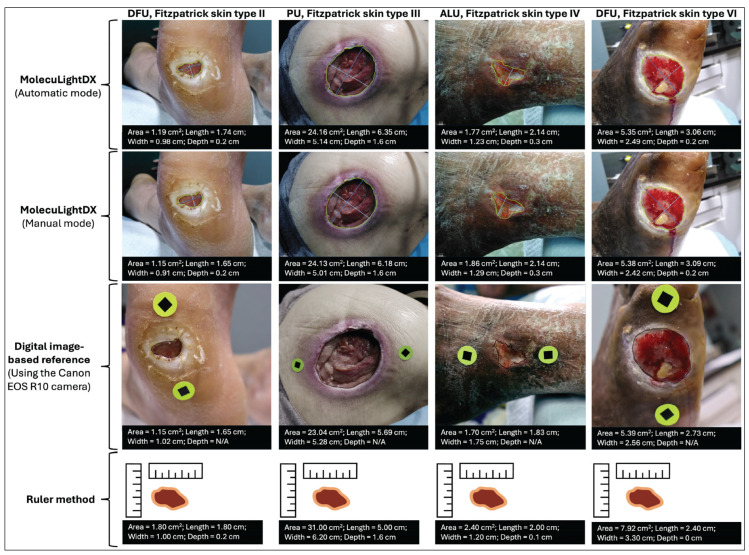
Illustrative examples of wound measurement using four different methods. Each column represents a distinct wound case, from left to right. **Column 1**: DFU, Fitzpatrick skin type II; **Column 2**: PU, Fitzpatrick skin type III; **Column 3**: ALU, Fitzpatrick skin type IV; and **Column 4**: DFU, Fitzpatrick skin type VI. For each wound, 4 measurement approaches are shown, from top to bottom: MolecuLightDX automatic mode, MolecuLightDX manual mode, a digital image-based reference using the Canon EOS R10 camera, and the ruler method. The top two rows display MolecuLightDX measurements with real-time readouts of wound surface area, length, width, and depth. The third row shows the digital image-based ground truth reference in which high-resolution images were captured using a Canon EOS R10 camera positioned 8–20 cm from the wound at a 90° angle with two 1 cm calibration stickers placed adjacent to the wound. Depth data were not available for these reference images, as they were used solely for surface area validation. The bottom row presents schematic representations of measurements derived from the ruler method, as corresponding clinical photographs were not collected for this standard-of-care technique. Together, the examples illustrate the visual and quantitative differences among measurement methods and highlight variations in wound morphology, type, and skin tone across study patients. Abbreviations: ALU, arterial ulcers; DFU, diabetic foot ulcers; and PU, pressure ulcers.

For the 33 clinical wounds evaluated, the mean percentage error relative to the digital image-based ground truth was 6.61% (SD: 6.14; 95% CI: 4.43–8.79) in automatic mode and 7.57% (SD: 5.31; 95% CI: 5.69–9.45) in manual mode. Representative images from this phase of the study are shown in Figure 3. The corresponding error for the ruler method was 77.90% (SD: 105.4; 95% CI: 40.53–115.27). Paired comparisons demonstrated statistically significant differences between both MolecuLightDX modes and the ruler method (*p* = 2.9 × 10^−5^ in automatic mode; *p* = 3.3 × 10^−5^ in manual mode). No significant difference was observed between MolecuLightDX’s automatic and manual modes (*p* = 0.337). Inter-user mean CVs were 0.053 (SD: 0.052; 95% CI: 0.034–0.071) for the automatic mode and 0.051 (SD: 0.026; 95% CI: 0.042–0.060) for manual mode. Intra-user CVs were 0.038 (SD: 0.021; 95% CI: 0.031–0.045) for automatic and 0.037 (SD: 0.012; 95% CI: 0.032–0.041) for manual mode. Intra- and inter-usability of manual and automatic measurement modes were below the acceptance threshold of 0.10.

#### Subgroup Analyses

Subgroup analyses were conducted across subgroups of skin tones, wound etiology, wound size, and wound duration using the MolecuLightDX only (Table 2). For Fitzpatrick skin type, the mean percentage error ranged 3.17–7.86% (SD range: 0.024–0.077) in the automatic mode and 6.02–8.14% (SD range: 0.024–0.064) in manual mode among six groups (Fitzpatrick I–VI), with *p* = 0.145. Inter-user CVs ranged 0.035–0.059 (SD range: 0.016–0.032) in automatic mode and 0.035–0.052 (SD range: 0.010–0.028) in manual mode. When stratified by wound type, the mean percentage error differed across the five groups (ALU, DFU, PU, VLU, and trauma) but was not powered to be statistically significant (*p* = 0.128). In automatic mode, the mean percentage error ranged from 3.22% in VLU to 9.36% in trauma wounds, with PU wounds showing the second-highest error (8.53%). In manual mode, the mean percentage error ranged from 5.85% in VLU to 11.45% in PU wounds, which exhibited the highest error in this mode. For wound size, the mean percentage error ranged 3.99–6.98% in automatic mode and 6.06–9.10% in manual mode among three groups (<2 cm^2^, 2–10 cm^2^, and >10 cm^2^), with *p* = 0.457. Bland–Altman plots showed a trend of increasing variability in larger wounds, though all values remained within the 10% error threshold. When stratified by wound duration, the mean percentage error differed across the four groups (<3 months, 3–6 months, 6–12 months, and >12 months) but was not powered to be statistically significant (*p* = 0.104). In automatic mode, the mean percentage error ranged from 2.53% in wounds of 3–6 months in duration to 7.99% in wounds >12 months old, with wounds <3 months also showing a relatively higher error (7.15%). In manual mode, the mean percentage error ranged from 4.43% in wounds of 3–6 months in duration to 8.25% in wounds <3 months old, while wounds >12 months similarly exhibited a higher error (8.16%). Generally, across all subgroups, mean CVs remained <10%, with a minor elevation in the inter-user CV of 0.107 observed for type IV in automatic mode.

### 3.3. AutoDepth Measurement

Seventeen 3D benchtop wound models representing DFU, VLU, and PU geometries were evaluated. True depths ranged from 0.13 cm to 2.78 cm. The mean absolute depth error for MolecuLightDX AutoDepth measurements was 0.87 mm (SD: 0.51; 95% CI: 0.80–0.93). Intra-user repeatability demonstrated an ICC of 0.999 (95% CI: 0.997–1) and inter-user repeatability ICC of 0.998 (95% CI: 0.996–0.999). Depth validation using 34 clinical wounds (depth range: 0.06–4.13 cm) yielded a mean absolute error of 0.98 mm (SD: 0.70; 95% CI: 0.89–1.06). Intra-user repeatability showed an ICC of 0.992 (SD: 0.984–0.996) and inter-user repeatability an ICC of 0.997 (SD: 0.994–0.998). The maximum depth validated in our testing was 4.1 cm, which aligns with the operational capability of the AutoDepth feature and the reference 3D rendering software (Artec Studio, Version 18.1.5.5). All AutoDepth measurements satisfied the predefined wound depth accuracy specification of ±2.0 mm (±0.2 cm), applied as the acceptance criterion. Intra- and inter-user ICC values exceeded the minimum reproducibility criterion of 0.90 (maximum possible: 1.0).

## 4. Discussion

The MolecuLightDX wound measurement device demonstrated high accuracy and reproducibility for both the wound surface area and depth assessments when compared with ruler-based and standard reference methods. The mean percentage error for surface area was <10%, representing an approximate tenfold improvement over geometric estimation with a paper ruler (77.9%). Notably, ruler-based measurements overestimated the wound area, consistent with previous reports [9,10,19]. For wound area, both intra- and inter-user mean CVs were <10%, and for AutoDepth (depth measurement), ICCs were approximately 0.99, indicating near-perfect reproducibility across wound types and skin tones. Collectively, these findings confirm that the MolecuLightDX device provides more accurate and consistent wound measurements than conventional ruler-based methods when compared with ground truth digital photography methods.

Accurate and reproducible wound surface area measurement, typically derived from length and width estimation, is a cornerstone of evidence-based wound care, with direct implications for clinical decision-making. Conventional ruler-based approaches assume regular wound geometry and rely heavily on operator judgment, making them particularly vulnerable to error in irregularly shaped wounds. Such variability can obscure subtle changes in wound size as it heals, delay treatment modification, and contribute to inconsistent clinical assessments. Even small geometric estimation errors in length and width measurements can distort perceived healing trajectories, potentially leading to inappropriate care decisions [8]. The marginally higher accuracy observed in benchtop wound models likely reflects controlled conditions, including clearly defined wound borders, standardized lighting, and stable imaging geometry, which collectively reduce segmentation ambiguity. In contrast, clinical wounds often exhibit irregular edges, exudate, or periwound discoloration that complicate boundary identification and require the subjective determination of wound orientation for length and width measurement. By providing automated, image-based wound boundary detection under standardized acquisition conditions, the MolecuLightDX mitigates the inherent subjectivity of ruler-based length and width measurement and supports more consistent, objective surface area assessment across diverse wound types.

Beyond surface area, the accurate assessment of wound depth is equally important [13], particularly for wounds with undermining or tunneling, where superficial closure may mask ongoing tissue loss. Traditional depth assessment methods, such as manual probing, are inherently operator-dependent and can be uncomfortable for patients, while also offering limited reproducibility. In this context, the near-perfect reproducibility observed for AutoDepth (ICC ≈ 0.99) underscores the value of contact-free, algorithm-driven depth measurement in supporting consistent assessments across users, wound types, and skin tones. By enabling standardized depth measurement without physical contact, AutoDepth addresses an important gap in routine wound assessment that is not reliably captured by ruler-based approaches.

To address both surface area and depth measurement challenges, digital wound measurement technologies have evolved to incorporate approaches such as structured-light projection, stereophotogrammetry, and 3D reconstruction [20]. Wu et al. [20] reviewed published articles on 12 wound measurement devices and categorized them into contact, partial-contact, and non-contact systems, each associated with strengths and limitations. Contact systems may deform the wound bed and cause discomfort, while partial-contact systems often require calibration stickers or reference markers and may have a limited depth measurement capability [20]. Non-contact systems demonstrate high reliability (ICC > 0.9) but can be bulky or operationally complex for routine bedside use [20]. Within this technological landscape, the MolecuLightDX combines the portability of handheld non-contact systems with workflow-integrated automation, eliminating the need for calibration stickers through a built-in optical rangefinder and using uniform illumination to reduce ambient-light variability at the point-of-care. These design features address several limitations noted by Wu et al. [20] and position the MolecuLightDX within the current generation of intelligent, clinically adaptable wound measurement devices.

This study was conducted under controlled clinical conditions at two centers within a single geographic region, which may limit the generalizability of findings. While real-world factors such as ambient lighting, device positioning, and user experience can introduce variability in practice, the MolecuLightDX mitigates many of these challenges through its integrated rangefinder, standardized illumination, and built-in image-quality checks (e.g., out-of-range distance, improper lighting, glare, or focus issues), helping ensure consistent image acquisition across different settings. The device is designed for wounds with clearly defined borders within its measurable size range and is not intended for circumferential wound assessment. Future investigations should evaluate how improved measurement precision influences clinical outcomes, including the prognostic value of surface area and depth changes over time.

Accurate and reproducible digital wound measurement supports important aspects of routine clinical workflow and treatment decision-making. In practice, wound measurements are frequently used to determine eligibility for advanced wound therapies such as cellular, acellular, and matrix-like products (CAMPs), where the accurate assessment of wound size helps ensure appropriate product selection and minimize material wastage. Standardized digital measurement can also facilitate the documentation of wound progression, including confirmation of insufficient wound area reduction prior to initiating advanced therapies and the monitoring of wound size reduction following treatment initiation within defined therapeutic windows (e.g., a ≥40% reduction in the wound surface area within 4 weeks is a reliable indicator of eventual complete healing [6]). While longitudinal tracking was not the focus of the study, the device is able to track changes in wound size and presents a percentage of the area change from the previous week for easy reference.

The portability of the MolecuLightDX device allows for its use across diverse care environments, including outpatient wound clinics, hospital settings, and mobile wound care services. In addition, the automated measurement workflow enables clinicians with varying levels of experience to obtain consistent wound measurements while reducing inter-operator variability and improving efficiency in busy clinical settings. Various design features ensure well-captured images that provide accurate measurements supporting its use outside of controlled environments and adaptability to real world conditions. The ability to rapidly obtain reliable wound measurements across a range of wound types supports the integration of digital measurement technologies into routine wound assessment workflows. Together, these features support the integration of standardized digital wound measurement into routine wound assessment workflows.

The integration of digital wound measurement data into electronic health records and decision-support systems allows for automated progress tracking, reduced assessment subjectivity, and strengthens adherence to evidence-based wound monitoring protocols. As the MolecuLightDX device operates within a secure digital platform, it is designed to integrate with EHR systems over secure wireless networks. Through this integration, wound images and measurement values are written directly into the patient’s chart, reducing duplicate data entry and improving workflow efficiency. These features support the secure storage and sharing of wound imaging data across members of the care team and facilitate the continuity of clinical documentation during multidisciplinary care and external review processes such as clinical or reimbursement audits. Future studies may also explore whether longitudinal digital wound measurements obtained with the MolecuLightDX device could contribute to predictive models of wound healing trajectories and treatment outcomes.

## 5. Conclusions

MolecuLightDX multimodal device incorporates highly accurate and consistent digital wound measurements, including contactless area and depth measurement, which was true across all skin types, wound sizes, and a variety of wound types. The device is significantly more accurate than the ruler method when compared to the ground truth. Collectively, these strengths support the adoption of standardized digital measurement in clinical workflows, enabling more consistent and objective monitoring of wound healing and more reliable documentation of wound dimensions in routine clinical practice.

## Figures and Tables

**Table 1:** Summary of accuracy and reproducibility results for benchtop and clinical testing of MolecuLightDX and ruler measurement techniques for wound area.

Measurement Technique	Wound Models	Clinical Wounds	Wound Models	Clinical Wounds	Wound Models	Clinical Wounds
	Mean Percentage Error (*p*-Value ^a^)		Inter-User CV		Intra-User CV	
MolecuLightDX (automatic)	2.57%	6.61% (*p* < 0.0001)	0.012	0.053	0.017	0.038
MolecuLightDX (manual)	3.34%	7.57% (*p* < 0.0001)	0.022	0.051	0.031	0.037
Ruler	N/A	77.90%	N/A	N/A	N/A	N/A

^a^ MolecuLightDX as compared to ground truth measurements. Maximum acceptable error = 10%; maximum acceptable inter- and intra-user CV = 0.1. Abbreviations: CV, coefficient of variation; N/A, not applicable.

**Table 2:** Mean percentage error and CV results for subgroup analyses using MolecuLightDX.

Subgroup	Range of Mean Percentage Error (SD Range)	*p*-Value	Range of CV (SD Range)	*p*-Value
Fitzpatrick skin type	Automatic mode: 3.17–7.86% (0.024–0.077) Manual mode: 6.02–8.14% (0.024–0.064)	0.145	Automatic mode: 0.035–0.059 (0.016–0.032) Manual mode: 0.035–0.052 (0.010–0.028)	0.987
Wound type	Automatic mode: 3.22–9.36% (0.029–0.100) Manual mode: 5.85–11.45% (0.031–0.062)	0.128	Automatic mode: 0.030–0.099 (0.019–0.101) Manual mode: 0.035–0.067 (0.007–0.033)	0.226
Wound size	Automatic mode: 3.99–6.98% (0.009–0.071) Manual mode: 6.06–9.10% (0.019–0.065)	0.457	Automatic mode: 0.026–0.057 (0.015–0.068) Manual mode: 0.032–0.055 (0.009–0.028)	0.831
Wound duration	Automatic mode: 2.53–7.99% (0.046–0.084) Manual mode: 4.43–8.25% (0.033–0.063)	0.104	Automatic mode: 0.038–0.061 (0.020–0.064) Manual mode: 0.036–0.051 (0.011–0.027)	0.356

Abbreviations: CV, coefficient of variation; SD, standard deviation.

## Data Availability

The datasets presented in this article are not readily available because the data contain commercially sensitive and proprietary information belonging to MolecuLight Inc. Requests to access the datasets should be directed to ljones@moleculight.com.
